# Functional Analysis of a Putative Target of Spatially Varying Selection in the *Menin1* Gene of *Drosophila melanogaster*

**DOI:** 10.1534/g3.118.200818

**Published:** 2018-11-07

**Authors:** Nicolas Svetec, Perot Saelao, Julie M. Cridland, Ary A. Hoffmann, David J. Begun

**Affiliations:** * Department of Evolution and Ecology, University of California, Davis, CA; † Laboratory of Evolutionary Genetics and Genomics, The Rockefeller University, New York, NY; ‡ School of BioSciences, Bio21 Institute, University of Melbourne, Australia

**Keywords:** DNA damage, chill coma recovery, local adaptation, latitudinal cline

## Abstract

While significant effort has been devoted to investigating the potential influence of spatially varying selection on genomic variation, relatively little effort has been devoted to experimental analysis of putative variants or genes experiencing such selection. Previous population genetic work identified an amino acid polymorphism in the *Mnn1* gene as one of the most strongly latitudinally differentiated SNPs in the genome of *Drosophila melanogaster* in the United States and Australia. Here we report the results of our transgenic analysis of this amino acid polymorphism. Genotypes carrying alternative *Mnn1* alleles differed in multiple phenotypes in a direction generally consistent with phenotypic differences previously observed along latitudinal clines. These results support inferences from earlier population genomic work that this variant influences fitness, and support the idea that the alleles exhibiting clines may be likely to have pleiotropic effects that are correlated along the axes favored by natural selection.

One of the defining problems of empirical population genetics is determining the genetic and population processes that explain the abundance of genetic variation in natural populations. In addressing this difficult problem, the discipline of population genetics has developed multiple intellectual traditions. These include the analysis of random samples of genetic variation to infer population genetic processes, and the experimental analysis of defined genetic variants to discover the properties of alleles that may influence or explain their spatial or temporal patterns in nature. In some cases these two intellectual traditions have coalesced, as in the classic work on the population genetics of lethal mutations in natural *Drosophila* populations (reviewed by Simmons and Crow 1977), the ecological genetics of Mendelian (or nearly Mendelian) traits (*e.g*., Ford 1965, Majerus 1998), or the ecological genetics of specific variants, including several that were identified via allozyme electrophoresis (*e.g*., Watt 1977, Hilbish and Koehn 1985, Chappell *et al.* 1988, Hochachka and Somero 1984). Population genomic analysis has occasionally been integrated with *in vivo* experimental work on specific candidate variants, however, such efforts have sensibly focused on genetically simple traits and alleles of large effect (*e.g.*, Shindo *et al.* 2005, Schmidt *et al.* 2008, Chan *et al.* 2010, Nadeau *et al.* 2016). While there are good examples of candidate targets of selection identified from allele frequency data that were later subjected to transgenic analysis in *D. melanogaster* (*e.g.*, Laurie-Ahlberg and Stam 1987, Catalán *et al.* 2016, Glaser-Schmitt and Parsch 2018, Yang and Edery 2018), the integration of population genomic inference with detailed *in vivo* functional analysis is still in its infancy.

Spatially varying selection is among the plausible selective mechanisms contributing to the maintenance of polymorphism in natural populations (*e.g.*, Levene 1953, Slatkin 1975, Felsenstein 1976, Endler 1977, Hedrick 1986, Yeaman 2015). For example, *D. melanogaster* phenotypic and genomic variation appears to be strongly influenced by recent selection in latitudinal clines in the United States and Australia (reviewed in Hoffmann and Weeks 2007, Adrion *et al.* 2015). Putative selected variants revealed by population genomic analysis of clinal samples in *D. melanogaster* (and in most species) are typically interpreted in one of two ways (*e.g.*, Turner *et al.* 2008, Kolaczkowski *et al.* 2011, Svetec *et al.* 2011, Fabian *et al.* 2012, Reinhardt *et al.* 2014, Machado *et al.* 2016, Božičević *et al.* 2016). First, variants or associated genes are subjected to enrichment analysis to generate hypotheses on the classes of sequence or phenotypes generally influenced by selection. Alternatively, the possible functional effects of such variants are speculated on based on inferences of gene function (related to annotations and expression patterns) and ecology. The quality of both types of hypotheses, however, may be compromised because of uncertainty in the identity of the true targets of selection and because the putative effects of candidate selected variants typically reflect gene annotations derived from laboratory mutations, the effects of which may differ substantially from those of natural variants. These uncertainties raise the question of the general role of experimental analysis of candidate targets of spatially varying selection for a model system such as *D. melanogaster*.

In this report we present our work using transgenic analysis to investigate a strong candidate SNP target of spatially varying selection as revealed by our previous population genetic analysis of latitudinal differentiation in *D. melanogaster* (Kolaczkowski *et al.* 2011, Reinhardt *et al.* 2014). Our focal variant is a non-synonymous SNP (nsSNP) in the *Mnn1* gene. The T/A (CAT to CAA) polymorphism at position *2L*:7061538 (FlyBase, v6.0) codes for a predicted Histidine(H)/Glutamine(Q) polymorphism at amino-acid residue 703 (H703Q). *Mnn1* has three transcripts RA, RB and RC; RB is unaffected by the focal SNP. All remaining *melanogaster* subgroup species reference sequences carry a Q at this residue, strongly suggesting that H is the derived allele. Indeed, all publicly available *Drosophila* reference sequences that include this residue are Q, with the exception of *D*. *bipectinata*, and *D. ananassae*, which are predicted to carry the H allele. The neighboring serine at amino-acid position 704 in *D. melanogaster* is predicted to be a phosphorylation site according to the NetPhos 2.0 software (Blom *et al.* 1999). Two alternative MNN1 protein alleles that differ only at the focal variant at residue H703Q have predicted phosphorylation probabilities at Ser704 of 0.69 (His allele) and 0.13 (Gln allele).

Our previous population genomic analyses of North America and Australia investigated allele frequency differentiation (*F_ST_*) between higher and lower latitude populations on both continents (Florida *vs.* Maine, and Queensland *vs.* Tasmania) (Kolaczkowski *et al.* 2011, Reinhardt *et al.* 2014). The goal of those analyses was to identify strongly differentiated variants potentially enriched for targets of spatially varying selection, in particular, those that showed substantial, parallel differentiation on both continents. Our focal nsSNP was among the more unusual variants as it was in the extreme tail of the nsSNP *F_ST_* distribution of both continents (0.33% and 0.8% tails of the North America and Australia *F_ST_* distributions, respectively), with the derived H allele occurring at higher frequency in higher latitude populations on both continents, consistent with strong, parallel selection. Other attributes of the SNP, such as the high degree of conservation of the corresponding residue and its location far from the breakpoints of any polymorphic cosmopolitan *D. melanogaster* inversions (the closest breakpoint, that of *In2R(NS)*, is more than 4 Mb away, Corbett-Detig and Hartl 2012) supported it as an attractive candidate target of spatially varying selection.

*Mnn1* is widely expressed in multiple tissues and developmental stages (FlyBase) and codes for a protein that is predicted to have histone-lysine N-methyltransferase activity. Phenotypic analysis of laboratory mutations has suggested a contribution of the gene to multiple traits, including DNA repair, generalized stress resistance, and development (Busygina *et al.* 2004, Papaconstantinou *et al.* 2005, Papaconstantinou *et al.* 2010). The DNA repair function drew our attention because of our work on the influence of spatially varying selection on embryo DNA repair (Svetec *et al.* 2016). Thus, we set out to use transgenic animals to investigate the influence of the focal SNP on an embryo DNA repair phenotype. However, unlike several of the candidate genes emerging from Svetec *et al.* (2016), most of which function specifically in DNA repair related phenotypes and were biased toward ovary or early embryo expression, *Mnn1* could plausibly affect many fitness components. Thus, this nsSNP was an interesting candidate not only to experimentally “validate” a population genomic outlier, but also to begin to estimate effect sizes and pleiotropic effects of such outliers. Therefore, to investigate the possible role of *Mnn1* variation in general stress response (Papaconstantinou *et al.* 2005), we also determined whether the focal variant influences response to physiological stressors (high and low temperature, starvation, and dessication) that might be relevant in natural populations distributed along a latitudinal gradient.

## Material and Methods

### Fly lines

The double balancer stock with genotype *w*; *Kr[If-1]/CyO*; *D[1]/TM3*, *Ser[1]* was obtained from the Bloomington Drosophila Stock Center (stock #7198). A stock carrying the *Mnn1* null allele, *Mnn1*^-e30^ (Papaconstantinou *et al.* 2005) was a gift from André Bédard’s lab. *D. melanogaster* Bloomington stock #9744 (genotype *y[1] w[1118]*; *PBac{y[+]-attP-9A}VK00027*) was used for the Pacman injections. All stocks were sib-mated for four generations prior to being used in crosses to reduce the amount of genetic variation segregating within balancer chromosome classes and on the *X* chromosome.

### Transgenic line construction

We used the Pacman recombineering resources (reviewed in Copeland *et al.* 2001) and followed the strategy detailed by Biswas *et al.* (2012) to generate our transgenic constructs. We first obtained the CH322-167K14 BAC chromosome containing the *Mnn1* gene region (*2L*:7,045,627-7,065,515 in *D. melanogaster* reference sequence version 6). This BAC also contains complete copies of genes *CG31907* and *Sem1*, as well as the 3′ end of *milt* upstream of *Mnn1*, and the 5′ end of *Nuf2* downstream of *Mnn1*. We confirmed by Sanger sequencing that the BAC contained the H allele, thus, we used recombineering in *E. coli* to engineer the Q allele. The region of interest was first replaced by a *galK* positive cassette, allowing successful recombinants to grow on selective media. A *galK* positive colony was then electroporated with a repair template to exchange the *galK* cassette for the *Mnn1* Q allele. Positive events (successful exchanges) were isolated by screening for *galK* negative colonies on minimal media and confirmed by Sanger sequencing. As the BAC backbone already contains mini-white and an *attB* site, using *PhiC31* recombinase, both alleles (H and Q) of the BAC chromosome could be directly inserted at the same *attP* site on chromosome 3 at cytological band 89E11 in *D*. *melanogaster* line #9744. Embryos from line #9744 were injected following standard procedures (BestGene Inc, Chino Hills CA, USA) and surviving adults were screened for the presence of red eye color reflecting successful insertion of a BAC carrying the mini-white transgene. Homozygous, true-breeding stocks of each transgene (MQ or MH) were established and then both were introgressed into the same *Mnn1* homozygous null background (Supplementary figure 1). The resulting lines, which were designated MQ and MH, were validated at the focal SNP by Sanger sequencing. Lines MQ and MH are expected to be isogenic except for the *Mnn1* H703Q variant. While we cannot rule out the possibility that low levels of balancer chromosome gene conversion resulted in other SNP differences between strains, such contamination should be random and unlikely to exhibit the large, directional effects on the phenotypes investigated here (below). Because experimental genotypes carried a non-functional endogenous copy of *Mnn1*, the transgenes were the only source of MNN1 protein for experimental animals.

### Population genetics

Allele frequencies from African and European samples were obtained from the Drosophila Genome NEXUS (Lack *et al.* 2015). Australian allele frequency estimates are from Kolaczkowski *et al.* (2011). Allele frequency estimates from Maine and Panama are from Reinhardt *et al.* (2014) and Svetec *et al.* (2016). New data from US samples are from Morven, Georgia (latitude 30.56N), Media, Pennsylvania (latitude 39.55N), and Middlefield, Connecticut (latitude 41.31). These derive from isofemale lines established by Paul Schmidt (University of Pennsylvania) in 2009. For each population, a pool of females was generated, one per line, from each population. The number of female flies used to create each pool were 21, 100, and 70 for Georgia, Pennsylvania, and Connecticut, respectively. Roughly 5 μg of DNA was used for paired-end sequencing library construction following the Illumina protocol. Fragmentation was done by sonication using the Diagenode Bioruptor at high power for 15 cycles of 30 sec on/30 sec off. The adapter-ligation product was gel-purified to select molecules ∼400 bp in length and then quantified using an Agilent Bioanalyzer. A total of 10 ng of size-selected ligation product was used as template for 10 cycles of library enrichment PCR. The enriched library was purified using Ampure XP beads (Beckman Coulter) and sequenced on a single lane of a flow cell with an Illumina GAIIx running Illumina software. Reads were aligned to version 6.04 of the *D. melanogaster* reference sequence using Bowtie2 with the–-very-sensitive setting. Variants were called using bcftools (samtools.github.io/bcftools) requiring a read quality score of 30 for inclusion. The mean coverage of US samples from Georgia, Pennsylvania, and Connecticut were 26.8, 25.9, and 29.7, respectively. We required a minimum of 20× coverage at a site in all populations and at least two observations of an alternate base call to consider a SNP in the analysis as described in Svetec *et al.* (2016). Raw data for these three new population samples can be found at the NCBI Short Read Archive under BioProject PRJNA495885.

### Egg hatch rate and UV sensitivity

UV sensitivity assays followed Svetec *et al.* (2016). For each transgenic line, we generated experimental animals by allowing groups of 10 to 15 parental flies to mate and lay eggs in a vial for 3-4 days. Those vials, which contained 4ml standard food, were placed into an incubator at 25° with 12:12 light/dark cycle and 50% humidity. The emerging offspring were anesthetized under light CO_2_ and placed into a new empty vial containing a small plastic spoon with dyed standard fly food. The spoon was changed every 24 hr for two days for egg laying habituation. In the morning of the first collection day, a new spoon with a drop of fresh yeast-water paste was placed into the vial. Two hours later, this first spoon was removed to discard previously retained eggs and then replaced by another spoon. Two hours later, groups of eggs (25-40 per replicate) were collected with a clean needle and delicately placed on a new spoon with fresh food. All eggs were placed on their side on the food surface, not touching any other egg. Potentially damaged eggs were discarded. Egg collection lasted 1 hr, after which spoons with eggs were distributed into two treatment groups: a control group which was left 60s on the bench and an experimental group which was immediately exposed to UV in an irradiator for 60s as described by Svetec *et al.* (2016). UVB incidence inside the irradiator was 201μW/cm^2^ and the temperature was maintained similar to the ambient temperature (23°; see Svetec *et al.* 2016 for details). Immediately after exposure, spoons were transferred to a vial with food, assigned a reference number so that scoring would be done blindly to genotype information, and then placed back into an incubator at 25° with 12:12 light:dark cycle and 50% humidity. Forty-eight hours later spoons were taken out of the vials and egg hatch was scored. UV sensitivity index was calculated as the difference between UV-exposed and UV-unexposed hatch rate. In other words, UV sensitivity is the reduction in hatch rate due to UV exposure (Svetec *et al.* 2016). UV sensitivity was calculated for each spoon and averaged for each genotype-by-treatment combination. Between 17 and 51 spoon replicates were used for each genotype and treatment for a total of 8392 embryos. Control hatch rate was measured for 3427 embryos.

### Chill coma recovery assays

Four-day-old virgin flies (12 males and 12 females) from each transgenic line (total 192 flies, four flies per vial) were subjected to cold stress for four time-durations (2, 4, 6, or 8 hr). Vials containing flies were placed on ice for the specified time and then placed back on a laboratory bench at room temperature (25°). The time required for recovery for each fly (fly assuming a normal stance) was recorded, as was survival at 24 and 48 hr after treatment. These assays are a proxy for cold tolerance.

### Desiccation resistance

Six-day-old flies (12 males and 12 females) from each transgenic line (four replicate vials of three flies each for each genotype and sex for a total of 48 flies) were subjected to desiccation stress (3 flies per vial). Vials were sealed with gauze and placed into a desiccation chamber containing silica beads. The chamber was sealed with petroleum jelly. The time until knockdown/death was recorded at hourly intervals until all flies had died.

### Heat shock

Five-day-old virgin flies (12 males and 12 females) from each transgenic line (total 48 flies) were subjected to heat stress by immersing flies in individual vials into a water bath set to 39° (without temperature ramping). The vial positions in the water bath were randomized. The time until knockdown was recorded.

### Starvation

Four-day-old virgin flies (12 males and 12 females) from each transgenic line were subjected to starvation stress (total 48 flies, 4 flies per vial). The flies were placed at 25° in a vial without media, sealed with gauze, and affixed end-to-end to a second vial containing wet cotton to provide moisture. The survival/death status was recorded at 4-hour intervals.

### Statistical analyses of phenotypic data

Statistical analyses were performed using R. As the data were unbalanced and departed from normality, type III ANOVAs were performed on both the untransformed values and the rank-transformed values of UV sensitivity index and chill coma recovery time. The untransformed and transformed data gave the same results (Supplementary Table 1); here we only report the results of the ANOVAs on rank-transformed values. Wilcoxon tests, Mann-Whitney *U*-tests, or proportional hazards survival analyses were used for other phenotypes.

### Data availability

New sequencing data from US *D. melanogaster* populations can be found in the NCBI Short Read Archive under BioProject PRJNA495885. Supplemental material available at Figshare: https://doi.org/10.25387/g3.7302002.

## Results

### Population genetics

**Figure 1:**
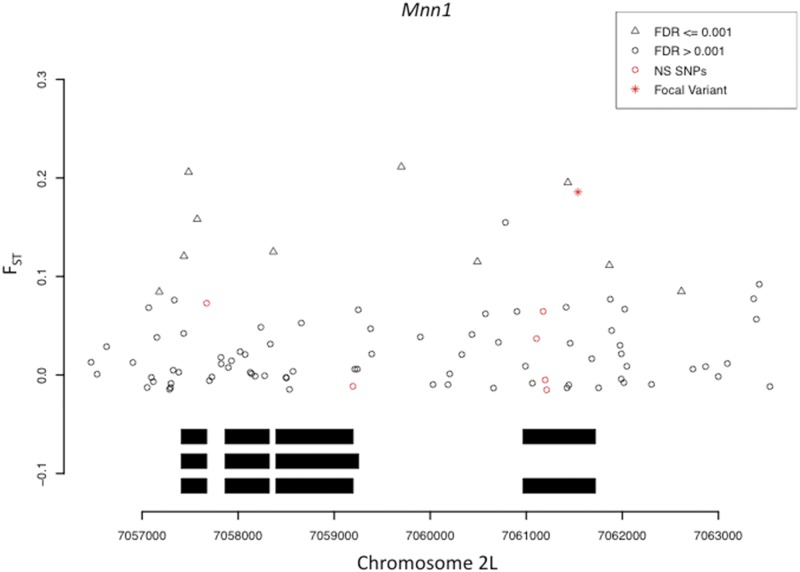
Estimates of genic SNP *F_ST_* in Maine *vs.* Panama for *Mnn1*. Coding sequence represented by black boxes (three isoforms). *F_ST_* estimates and false discovery rates (FDR) follow those from Svetec *et al.* (2016). The focal variant is the most differentiated nsSNP in the gene.

As aforementioned, while our initial interest in this SNP was driven by our analysis of substantial, parallel geographic differentiation in the Americas and Australia, additional data, including more extensive sampling of the Americas and Africa have accumulated since that time. Thus, we carried out a population genetic analysis aimed at a more comprehensive characterization of allele frequency variation in the *Mnn1* gene region. Reinhardt *et al.* (2014) identified the focal SNP as an *F_ST_* outlier in the US and Australia (Reinhardt *et al.* 2014). To compare those results with differentiation between Maine and a neotropical population, we estimated *F_ST_* of the focal variant between Maine and Panama (Svetec *et al.* 2016) and found that it was in the 3% tail of the nsSNP distribution, consistent with our Maine *vs.* Florida estimate, though somewhat less differentiated. Figure 1 shows that the focal SNP, while not the only strongly differentiated *Mnn1* SNP between Maine and Panama, is substantially more differentiated than other nsSNPs in the gene. We extended these results by characterizing patterns of geographic variation in additional population samples from the US and from Africa and Europe.

**Figure 2:**
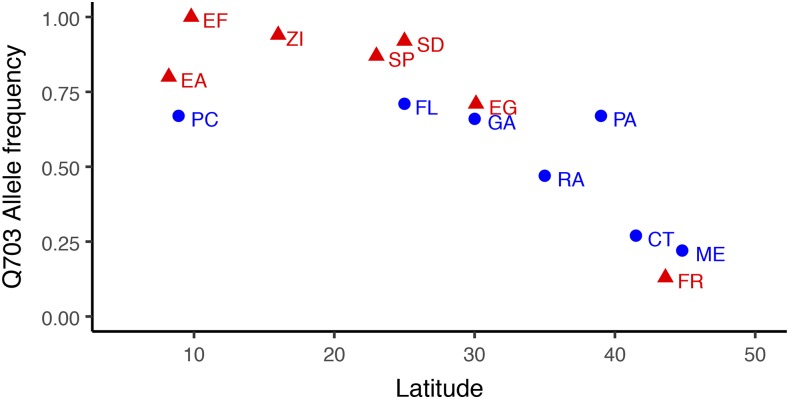
Frequencies of the *Mnn1* Q allele in Old World and New World samples: In blue is the New World cline: Panama (PC), Florida (FL), Georgia (GA), N. Carolina (RAL), Pennsylvania (PA), Connecticut (CT), Maine (ME). In red is the Old World cline: Ethiopia low altitude (EA), Ethiopia high altitude: 3070m (EF), Zimbabwe (ZI), South Africa low altitude (SP), South Africa high altitude: 2000m (SD), Egypt (EG), France (FR)

Figure 2 shows that the focal SNP exhibits strong latitudinal differentiation in Old World and New World samples. Linear regression of H703Q allele frequency *vs.* latitude led to R^2^= 0.47 (*P* = 0.089) and R^2^ = 0.67 (*P* = 0.025) for the New World and Old World, respectively. Fisher’s combined probability for the two regions rejects the null hypothesis of no correlation between allele frequency and latitude (*P* = 0.016). Linear regression on the combined data were also highly significant (R^2^ = 0.63, *P* < 0.001). Overall, the data in Figure 2 strongly support the hypothesis that the H703Q polymorphism is clinal. Moreover, the presence of latitudinal clines in both Old World and New World populations supports the idea that the clines result from selection in both the ancestral and recently established regions, rather than demographic processes in recently established populations.

### UV sensitivity index

**Figure 3:**
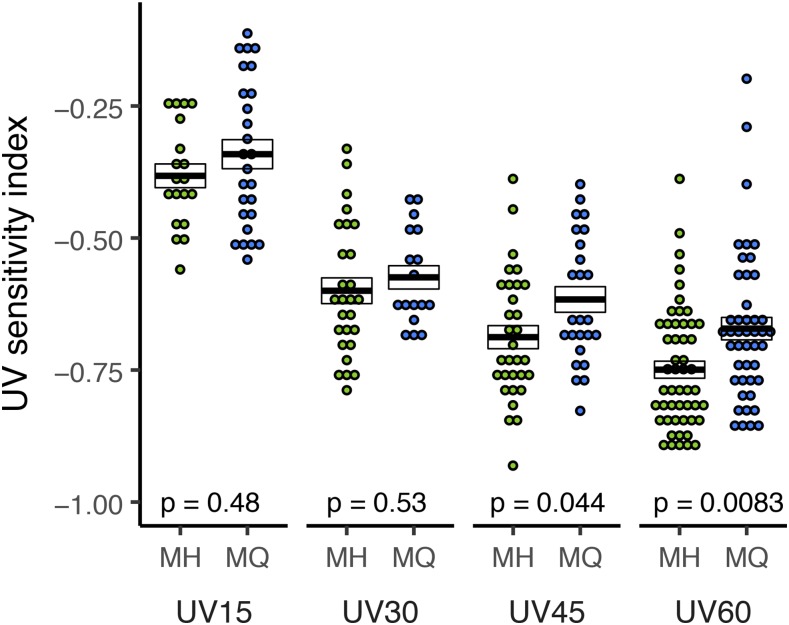
Early embryo UV sensitivity: The full model ANOVA with Genotype and UV treatment as factors was statistically significant (df = 7, 233; *F* = 32.62; *P* < 0.001). *Mnn1* (H703Q) contributes to variation in UV sensitivity as the Genotype factor was significant (df = 1; *F* = 7.90; *P* = 0.005). UV exposure also had a significant effect (df = 3; *F* = 68.60; *P* < 0.001); the interaction was not significant. UV sensitivity index (corresponding to the reduction in embryo hatch rate due to UV exposure) was measured for 1-3 hr. old embryos carrying either of the two *Mnn1* alleles. Embryos were exposed to UVB for 15, 30, 45, and 60 sec. Each data point represents the UV sensitivity index of a pool of 25 to 40 embryos. Sample size range is 17 ≤ n ≤ 51 embryo sets. Crossbars represent mean ± SE and p-values correspond to a Wilcoxon test on MH *vs.* MQ for each UV treatment. UV15: W = 363.5; *P* = 0.482; UV30 W = 256; *P* = 0.530; UV45 W = 510; *P* = 0.044 UV60 W = 1507; *P* = 0.008. At the 60 sec dose the H allele reduces UV sensitivity by about 12%.

In Svetec *et al.* (2016) we presented several lines of evidence in support of the idea that natural populations of *D. melanogaster* experience spatially varying selection on embryonic DNA repair resulting from latitudinal variation in UV incidence. First, we demonstrated that lower latitude embryos tend to be more resistant to UV exposure than higher latitude embryos. We further demonstrated that embryo UV resistance is correlated with oocyte repair of damaged sperm DNA. Finally, we observed latitudinal differentiation for early embryo transcript abundance and SNPs associated with DNA repair genes. In spite of the strong correlational support for our hypothesis, our previous work presented no direct evidence of a strongly differentiated variant affecting embryo DNA repair. Figure 3 shows that the Q allele, which is more common in higher UV environments, consistently exhibits greater mean resistance to UV.

### Egg hatch rate

**Figure 4:**
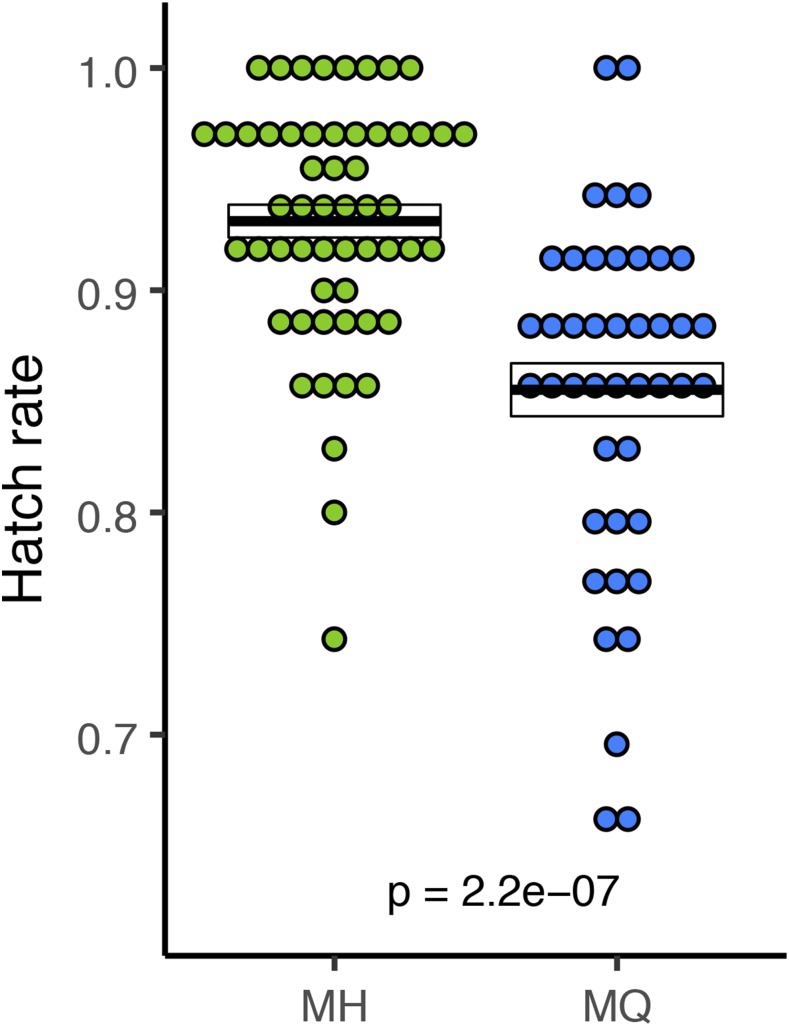
Egg hatch rate. Each point represents the hatch rate of a set of 25 to 40 embryos. Sample sizes are n = 55, n = 43 embryo sets, for MH and MQ respectively. Crossbars represent mean ± SE with p-value corresponding to a Wilcoxon test on MH *vs.* MQ.

Svetec *et al.* (2016) showed that in addition to a cline for UV sensitivity, there is a countervailing cline for embryo viability (in the absence of UV) such that lower latitude populations of *D. melanogaster* had reduced egg hatch rates compared to higher latitude populations. Svetec *et al.* (2016) speculated that these countervailing clines might represent a DNA-repair related trade-off such that variants conferring increased embryo DNA repair capacity have reduced hatch rates in the absence of UV. Such trade-offs could, in principle, contribute to the maintenance of variation. Our direct test of the focal SNP supports this hypothesis (Figure 4), as the allele more common in high-UV environments exhibits a reduced intrinsic hatch rate (mean ± SE: 0.931 ± 0.007; n = 55 for the H allele and 0.855 ± 0.011; n = 43 for the Q allele; Mann-Whitney U = 462; *P* < 0.001). The H allele increases embryo viability by about 9%.

### Chill coma recovery

**Figure 5:**
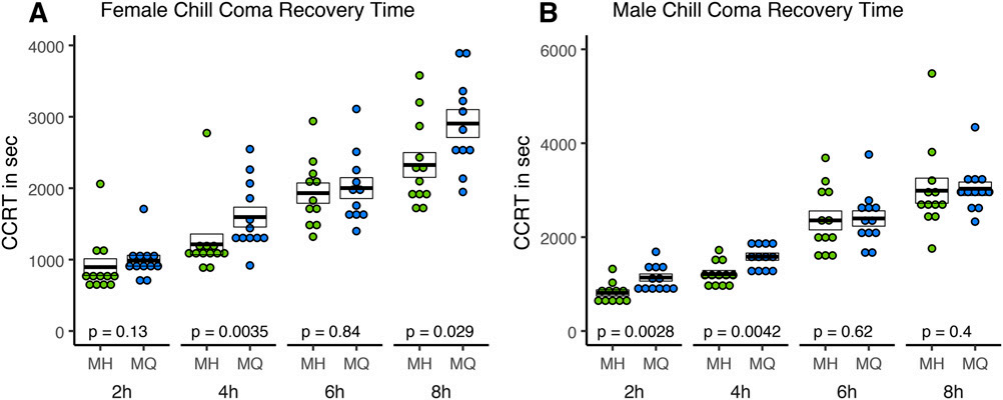
Chill coma recovery time: Phenotypes were measured for adult female (A) and male (B) flies exposed to cold for 2, 4, 6, and 8 hr. Each point depicts the recovery time (stand up) from cold shock. Crossbars represent mean ± SE, with p-values corresponding to a Wilcoxon test on MH *vs.* MQ for each cold treatment. Sample sizes are n = 11 or n = 12 vials.

The full model ANOVA with genotype, sex and treatment as factors was significant (df = 15, 172; *F* = 39.48; *P* < 0.001). The transgenic lines showed different response to cold treatment, as the Genotype factor was statistically significant (df = 1; *F* = 20.47; *P* < 0.001) with MH, the high latitude allele, recovering more quickly than MQ (Figure 5). The effect size varies considerably between sexes and among treatments, with the greatest effect sizes being 40% for males in the 2-hr. treatment and 31% for females in the 4-hr. treatment. Both Sex (df = 1; *F* = 7.71; *P* = 0.006) and Treatment (df = 3; *F* = 183.56; *P* < 0.001) terms were significant, with females recovering faster than males, and longer cold exposures correlated with longer recovery times. We observed no significant interactions between Sex and Treatment factors.

### Desiccation and heat shock resistance

**Figure 6:**
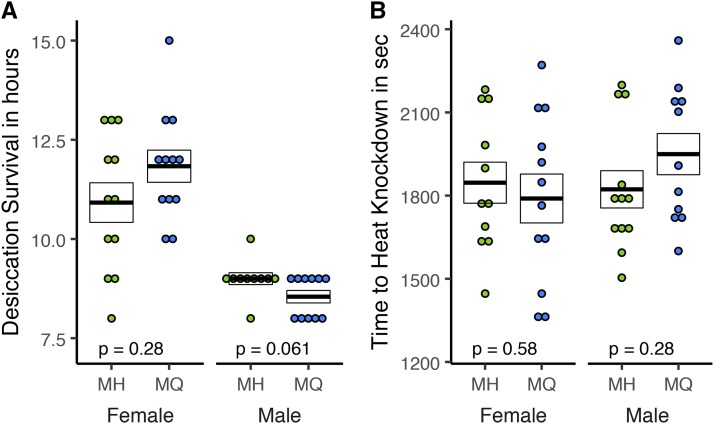
Desiccation and heat shock resistance were measured for adult female and male flies. For desiccation resistance (A) each data point represents the time in hours until death. For heat shock resistance (B), each data point represents the mean time in hours until death of all individuals in a vial. For both traits, sample sizes are 10 ≤ n ≤12, crossbars represent mean ± SE and p-values correspond to a Wilcoxon test on MH *vs.* MQ for each sex.

We used proportional hazards fit to analyze our measurements of desiccation resistance. As expected (*e.g.*, Hoffmann and Parsons 1989), females were significantly more tolerant to desiccation (*P* < 0.001) than males but we did not detect a significant effect of genotype (Figure 6A) (*P* = 0.76). Similarly, we found no significant differences between genotypes for heat tolerance (Figure 6B): MH *vs.* MQ: Males: Mann-Whitney *U* = 48; *P* = 0.28 and females: Mann-Whitney *U* = 56.5; *P* = 0.57.

### Starvation resistance

**Figure 7:**
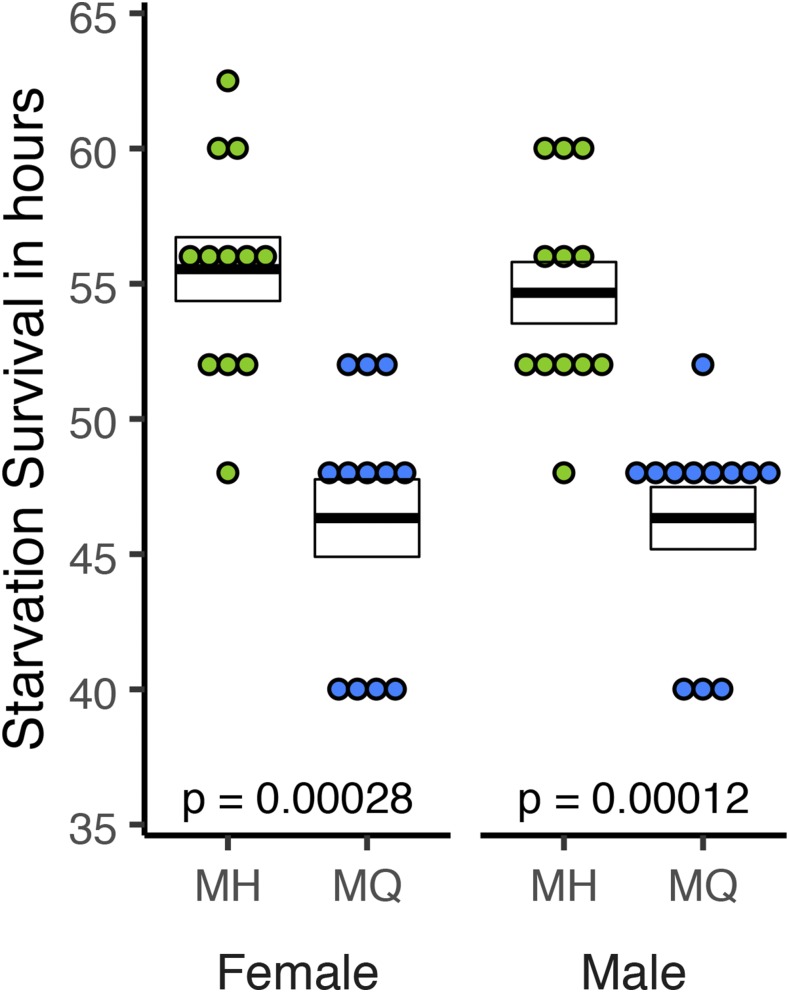
Starvation resistance was measured for adult female and male flies. Each point represents the time in hours until the death of one individual. Crossbars represent mean ± SE; p-values correspond to a Wilcoxon test on MH *vs.* MQ for each sex. Sample sizes are all n = 12.

We used a proportional hazards fit analysis to compare the starvation resistance of the MQ an MH genotypes. We found a significant effect of Genotype (*P* < 0.001 for both sexes), but no effect of Sex or Genotype-by-Sex. This result was consistent with a re-analysis using Wilcoxon tests on the same data sorted by sex (Figure 7). The MH allele increased survival under starvation by 20% and 18% for females and males, respectively (*P* < 0.001 for both sexes).

## Discussion

Given the polygenic nature of fitness variation, one might reasonably expect that the functional analysis of individual candidate selected variants inferred from population genetic data may often fail to yield experimental evidence of phenotypic effects as a result of low power. An alternative hypothesis for negative experimental results on such candidates is that they are population genetic false positives or that the appropriate phenotypes have not been measured. The positive experimental results reported here are cause for a somewhat more optimistic view, in support of other similar work on Drosophila (Laurie-Ahlberg and Stam 1987, Catalán *et al.* 2016, Siddiq *et al.* 2017, Glaser-Schmitt and Parsch 2018, Yang and Edery 2018). We compared two genotypes that differed for a single amino acid variant and found that four of six assayed phenotypes were different: embryo UV sensitivity, embryo hatch rates, chill coma recovery, and starvation resistance (Table 1). Importantly, for embryo UV sensitivity, hatch rates, and chill coma recovery, the direction of the observed allelic effect was consistent with general patterns of phenotypic differentiation between high and low latitude populations and with the frequency differences of alternative alleles at the focal SNP. Thus, the pleiotropic effects of the alleles appear to generally be in the same direction as the mean population differences (Lovell *et al.* 2013). For starvation resistance, however, we observed that the high latitude allele was more resistant, while an analysis of Australian populations (Hoffmann *et al.* 2001) revealed that low latitude populations were slightly, though significantly, more starvation resistant. However, there are few studies of geographic variation in starvation resistance for *D. melanogaster* (Goenaga *et al.* 2013, Božičević *et al.* 2016) and the relationship between geography and starvation resistance may be weak (Brown *et al.* 2018).

**Table 1:** Summary of phenotypic effects of the H (high latitude) and Q (low latitude) alleles of *Mnn1*

Life stage	Phenotype			
Embryo	Control hatch rate	MH	>	MQ
	UV sensitivity index	MH	<	MQ
Adult	Chill coma recovery	MH	>	MQ
	Heat shock tolerance	MH	=	MQ
	Starvation resistance	MH	>	MQ
	Desiccation resistance	MH	=	MQ

An apparent puzzle generated by these data is that the effect sizes seem surprisingly large. For example, focusing on UV sensitivity index at the 60 sec dose, which was the same treatment used in our earlier experiments (Svetec *et al.* 2016) and assuming the genetic background used for the recombineering experiments was more temperate-like than tropical-like, we observe that the MH genotype has a UV sensitivity index (-0.74) nearly identical to the mean value for the Rhode Island + Maine populations (-0.76). However, introducing the focal amino acid change leads to a phenotype (UV sensitivity index = -0.67) that is similar to the mean value previously observed for Florida + Mexico (-0.67), corresponding to a genotypic effect of H703Q on UV sensitivity of about 0.035 units. While this effect is substantial, it does not imply that the influence of the focal SNP explains most of the mean population difference. For example, given the frequency differences for the Q allele between Rhode Island and Florida in Figure 2 and assuming additivity, the contribution of the frequency difference of the H703Q polymorphism to the difference in population means is about 0.015 UV sensitivity units (Falconer 1989). This is consistent with a complex, though not highly polygenic basis for population differences in UV sensitivity.

Our results support the view that the transgenic analysis of major population genomic outliers, which are expected to be among the most strongly selected variants in the genome, will be a valuable approach for integrating studies of effect size and pleiotropic effects on specific phenotypes, with estimates of fitness effects indirectly inferred from population genetic analysis. Whether such an experimental program can succeed on a substrate of less extreme population genomic outliers is an open question. A longstanding and remaining problem for Drosophila population genetics (and empirical population genetics in most organisms) is that while fitness effects can be indirectly estimated from population genetic data and effects of individual variants on particular phenotypes can, in principle, be estimated in the laboratory or in semi-natural populations (*e.g.*, Mitrovski and Hoffmann 2001), the measurement of fitness effects of individual variants in a manner that reflects their properties in natural genotypes in natural populations is difficult, if not impossible. It is plausible that even variants with major phenotypic effects in laboratory experiments, such as the focal SNP investigated here, may have small fitness effects in natural populations, either because of their distributions of pleiotropic effects on both measured and unmeasured traits, or because of the properties of interactions between natural genotypes and natural biotic or abiotic variation.

## References

[bib1] Adrion J. R. Hahn M. W. Cooper B. S., 2015 Revisiting classic clines in Drosophila melanogaster in the age of genomics. Trends Genet. 31: 434–444. 10.1016/j.tig.2015.05.006 26072452 PMC4526433

[bib2] Biswas K. Stauffer S. Sharan S. K., 2012 Using recombineering to generate point mutations:galK-based positive–negative selection method, pp. 121–131 in Gene Synthesis: Methods and Protocols, edited by J. Peccoud, Humana Press, New York 10.1007/978-1-61779-564-0_10 PMC6668620 22328430

[bib3] Blom N. Gammeltoft S. Brunak S., 1999 Sequence and structure-based prediction of eukaryotic protein phosphorylation sites. J. Mol. Biol. 294: 1351–1362. 10.1006/jmbi.1999.3310 10600390

[bib4] Božičević V. Hutter S. Stephan W. Wollstein A., 2016 Population genetic evidence for cold adaptation in European *Drosophila melanogaster* populations. Mol. Ecol. 25: 1175–1191. 10.1111/mec.13464 26558479

[bib5] Brown E. B. Torres J. Bennick R. A. Rozzo V. Kerbs A., 2018 Variation in sleep and metabolic function Is associated with latitude and average temperature in *Drosophila melanogaster*. Ecol. Evol. 8: 4084–4097. 10.1002/ece3.3963 29721282 PMC5916307

[bib6] Busygina V. Suphapeetiporn K. Marek L. R. Stowers R. S. Xu T., 2004 Hypermutability in a Drosophila model for Multiple Endocrine Neoplasia Type 1. Hum. Mol. Genet. 13: 2399–2408. 10.1093/hmg/ddh271 15333582

[bib7] Catalán A. Glaser-Schmitt A. Argyridou E. Duchen P. Parsch J., 2016 An indel polymorphism in the MtnA 3′ untranslated region is associated with gene expression variation and local adaptation in *Drosophila melanogaster*. PLoS Genet. 12: e1005987 10.1371/journal.pgen.1005987 27120580 PMC4847869

[bib44] Chan Y. F. Marks M. E. Jones F. C. Villarreal G. Shapiro M. D., 2010 Adaptive evolution of pelvic reduction in sticklebacks by recurrent deletion of a Pitx1 enhancer. Science. 327: 302–305. 10.1126/science.1182213 PMC3109066 20007865

[bib8] Chappell M. A. Hayes J. P. Snyder L. R. G., 1988 Hemoglobin polymorphisms in deer mice (*Peromyscus maniculatus*): physiology of beta-globin variants and alpha-globin recombinants. Evolution 42: 681–688. 10.1111/j.1558-5646.1988.tb02486.x 28563877

[bib9] Copeland N. G. Jenkins N. A. Court D. L., 2001 Recombineering: A Powerful New Tool for Mouse Functional Genomics. Nat. Rev. Genet. 2: 769–779. 10.1038/35093556 11584293

[bib10] Corbett-Detig R. B. Hartl D. L., 2012 Population genomics of inversion polymorphisms in *Drosophila melanogaster*. PLoS Genet. 8: e1003056 (erratum: PLoS Genet. 9: 10.1371/annotation/b1cace11-17ed-456e-b8a9-006c09125bd0) 10.1371/journal.pgen.1003056 23284285 PMC3527211

[bib11] Endler J. A., 1977 *Geographic Variation*, *Speciation*, *and Clines*, Princeton University Press, Princeton, NJ. 409931

[bib12] Fabian D. K. Kapun M. Nolte V. Kofler R. Schmidt P. S., 2012 Genome-wide patterns of latitudinal differentiation among populations of *Drosophila melanogaster* from North America. Mol. Ecol. 21: 4748–4769. 10.1111/j.1365-294X.2012.05731.x 22913798 PMC3482935

[bib45] Falconer D. S., 1989 Introduction to Quantitative Genetics. 3rd Edition, Longman Scientific and Technical, New York.

[bib13] Felsenstein J., 1976 The theoretical population genetics of variable selection and migration. Annu. Rev. Genet. 10: 253–280. 10.1146/annurev.ge.10.120176.001345 797310

[bib46] Ford E. B., 1965 Genetic Polymorphism. M.I.T Press, Cambridge, MA.

[bib14] Glaser-Schmitt A. Parsch J., 2018 Functional characterization of adaptive variation within a cis-regulatory element influencing *Drosophila melanogaster* growth. PLoS Biol. 16: e2004538 10.1371/journal.pbio.2004538 29324742 PMC5783415

[bib15] Goenaga J. Fanara J. J. Hasson E., 2013 Latitudinal variation in starvation resistance is explained by lipid content in natural populations of *Drosophila melanogaster*. Evol. Biol. 40: 601–612. 10.1007/s11692-013-9235-6

[bib16] Hedrick P. W., 1986 Genetic polymorphism in heterogeneous envi- ronments: a decade later. Annu. Rev. Ecol. Syst. 17: 535–566. 10.1146/annurev.es.17.110186.002535

[bib17] Hilbish T. J. Koehn R. K., 1985 The physiological basis of selection at the LAP locus. Evolution 39: 1302–1317. 10.1111/j.1558-5646.1985.tb05696.x 28564261

[bib18] Hochachka P. W. Somero G. N., 1984 Biochemical Adaptation, Princeton University Press, Princeton, NJ 10.1515/9781400855414

[bib19] Hoffmann A. A. Parsons P. A., 1989 An integrated approach to environmental stress tolerance and life history variation: desiccation tolerance in Drosophila. Biol. J. Linn. Soc. Lond. 37: 117–136. 10.1111/j.1095-8312.1989.tb02098.x

[bib20] Hoffmann A. A. Hallas R. Sinclair C. Mitrovski P., 2001 Levels of variation in stress resistance in Drosophila among strains, local populations, and geographic regions: Patterns for desiccation, starvation, cold resistance, and associated traits. Evolution 55: 1621–1630. 10.1111/j.0014-3820.2001.tb00681.x 11580021

[bib21] Hoffmann A. A. Weeks A. R., 2007 Climatic selection on genes and traits after a 100 year-old invasion: A critical look at the temperate-tropical clines in *Drosophila melanogaster* from eastern Australia. Genetica 129: 133–147. 10.1007/s10709-006-9010-z 16955331

[bib22] Kolaczkowski B. Kern A. D. Holloway A. K. Begun D. J., 2011 Genomic differentiation between temperate and tropical Australian populations of *Drosophila melanogaster*. Genetics 187: 245–260. 10.1534/genetics.110.123059 21059887 PMC3018305

[bib23] Lack J. B. Cardeno C. M. Crepeau M. W. Taylor W. Corbett-Detig R. B., 2015 The Drosophila Genome Nexus: A population genomic resource of 623 *Drosophila melanogaster* genomes, including 197 from a single ancestral range population. Genetics 199: 1229–1241. 10.1534/genetics.115.174664 25631317 PMC4391556

[bib24] Laurie-Ahlberg C. Stam L., 1987 Use of P-Element-mediated transformation to identify the molecular basis of naturally occurring variants affecting *Adh* expression in *Drosophila melanogaster*. Genetics 115: 129–140. 2881843 10.1093/genetics/115.1.129 PMC1203048

[bib25] Levene H., 1953 Genetic equilibrium when more than one ecological niche is available. Am. Nat. 87: 331–333. 10.1086/281792

[bib47] Lovell J. Juenger T. Michaels S. Lasky J. Platt A., 2013 Pleiotropy of FRIGIDA enhances the potential for multivariate adaptation Proceedings of the Royal Society B: Biological Sciences 280: 20131043–20131043. 10.1098/rspb.2013.1043 PMC3774242 23698015

[bib26] Machado H. Bergland A. O. O’Brien K. R. Behrman E. L. Schmidt P. S., 2016 Comparative population genomics of latitudinal variation in *Drosophila simulans* and *Drosophila melanogaster*. Mol. Ecol. 25: 723–740. 10.1111/mec.13446 26523848 PMC5089931

[bib27] Majerus M. E. N., 1998 Melanism: Evolution in Action, Oxford University Press, Oxford, United Kingdom.

[bib28] Mitrovski P. Hoffmann A. A., 2001 Postponed reproduction as an adaptation to winter conditions in *Drosophila melanogaster*: evidence for clinal variation under semi-natural conditions. Proc. Biol. Sci. 268: 2163–2168. 10.1098/rspb.2001.1787 11600081 PMC1088861

[bib29] Nadeau N. J. Pardo-Diaz C. Whibley A. Supple M. A. Saenko S. V., 2016 The gene cortex controls mimicry and crypsis in butterflies and moths. Nature 534: 106–110. 10.1038/nature17961 27251285 PMC5094491

[bib30] Papaconstantinou M. Pepper A. N. Wu Y. Kasimer D. Westwood T. 2010 Menin links the stress response to genome stability in *Drosophila melanogaster* PLoS ONE 5 (11). Public Library of Science: e14049 10.1371/journal.pone.0014049 PMC2987805 21124979

[bib31] Papaconstantinou M. Wu Y. Pretorius H. N. Singh N. Gianfelice G., 2005 *Menin* is a regulator of the stress response in *Drosophila melanogaster*. Mol. Cell. Biol. 25: 9960–9972. 10.1128/MCB.25.22.9960-9972.2005 16260610 PMC1280255

[bib32] Reinhardt J. A. Kolaczkowski B. Jones C. D. Begun D. J. Kern A. D., 2014 Parallel geographic variation in *Drosophila melanogaster*. Genetics 197: 361–373. 10.1534/genetics.114.161463 24610860 PMC4012493

[bib33] Schmidt P. S. Zhu C.-T. Das J. Batavia M. Yang L., 2008 An amino acid polymorphism in the couch potato gene forms the basis for climatic adaptation in *Drosophila melanogaster*. Proc. Natl. Acad. Sci. USA 105: 16207–16211. 10.1073/pnas.0805485105 18852464 PMC2570987

[bib34] Shindo C. M. J. Aranzana C. Lister C. Baxter C. Nicholls C., 2005 Role of FRIGIDA and FLOWERING LOCUS C in determining variation in flowering time of Arabidopsis. Plant Physiol. 138: 1163–1173. 10.1104/pp.105.061309 15908596 PMC1150429

[bib35] Siddiq M. Loehlin D. W. Montooth K. L. Thornton J. W., 2017 Experimental test and refutation of a classic case of molecular adaptation in *Drosophila melanogaster*. Nat. Ecol. Evol. 1: 0025 10.1038/s41559-016-0025 PMC5703048 28812605

[bib36] Simmons M. J. Crow J. F., 1977 Mutations affecting fitness in Drosophila populations. Annu. Rev. Genet. 11: 49–78. 10.1146/annurev.ge.11.120177.000405 413473

[bib37] Slatkin M., 1975 Gene flow and selection in a two-locus system. Genetics 81: 787–802. 1213276 10.1093/genetics/81.4.787 PMC1213435

[bib38] Svetec N. Werzner A. Wilches R. Pavlidis P. Álvarez-Castro J. M., 2011 Identification of X–Linked quantitative trait loci affecting cold tolerance in *Drosophila melanogaster* and fine mapping by selective sweep analysis. Mol. Ecol. 20: 530–544. 10.1111/j.1365-294X.2010.04951.x 21199023 PMC3668310

[bib39] Svetec N. Cridland J. M. Zhao L. Begun D. J., 2016 The adaptive significance of natural genetic variation in the DNA damage response of *Drosophila melanogaster*. PLoS Genet. 12: e1005869 10.1371/journal.pgen.1005869 26950216 PMC4780809

[bib40] Turner T. L. Levine M. T. Eckert M. L. Begun D. J., 2008 Genomic analysis of adaptive differentiation in *Drosophila melanogaster*. Genetics 179: 455–473. 10.1534/genetics.107.083659 18493064 PMC2390623

[bib41] Watt W. B., 1977 Adaptation at Specific Loci. I. Natural selection on phosphoglucose isomerase of Colias butterflies: Biochemical and population aspects. Genetics 87: 177–194. 914029 10.1093/genetics/87.1.177 PMC1213725

[bib42] Yang Y. Edery I., 2018 Parallel clinal variation in the mid-day siesta of *Drosophila melanogaster* implicates continent-specific targets of natural selection. PLoS Genet. 14: e1007612 10.1371/journal.pgen.1007612 30180162 PMC6138418

[bib43] Yeaman S., 2015 Local adaptation by alleles of small effect. Am. Nat. 186: S74–S89. 10.1086/682405 26656219

